# Comparative Study of Steel-FRP, FRP and Steel-Reinforced Coral Concrete Beams in Their Flexural Performance

**DOI:** 10.3390/ma13092097

**Published:** 2020-05-01

**Authors:** Lei Wang, Jiwang Zhang, Changshi Huang, Feng Fu

**Affiliations:** 1 College of Civil Engineering and Architecture, Guilin University of Technology, Guilin 541004, China; wanglei@glut.edu.cn (L.W.); yataozhao@glut.edu.cn (J.Z.); 2 Guangxi Beibu Gulf Engineering Research Center for Green Marine Materials, Guilin 541004, China; 2017040@glut.edu.cn; 3 School of Mathematics, Computer Science and Engineering, City, University of London, London EC1V 0HB, UK

**Keywords:** coral concrete beam, flexural performance, CFRP bars, steel–CFRP composite bars (SCFCB), SCFCB, lightweight aggregate concrete

## Abstract

In this study, a comparative study of carbon fiber reinforced polymer (CFRP) bar and steel–carbon fiber composite bar (SCFCB) reinforced coral concrete beams was made through a series of experimental tests and theoretical analyses. The flexural capacity, crack development and failure modes of CFRP and SCFCB-reinforced coral concrete were investigated in detail. They were also compared to ordinary steel-reinforced coral concrete beams. The results show that under the same conditions of reinforcement ratios, the SCFCB-reinforced beams exhibit better performance than CFRP-reinforced beams, and stiffness is slightly lower than that of steel-reinforced beams. Under the same load conditions, the crack width of SCFCB beams was between that of steel-reinforced beams and CFRP bar-reinforced beams. Before the steel core yields, the crack growth rate of SCFCB beam is similar to the steel-reinforced beams. SCFCB has a higher strength utilization rate—about 70–85% of its ultimate strength. Current design guidance was also examined based on the test results. It was found that the existing design specifications for FRP-reinforced normal concrete is not suitable for SCFCB-reinforced coral concrete structures.

## 1. Introduction

Coral concrete has been widely used in marine engineering. As it is produced using coral debris and seawater, it can also solve the problem of material shortage in ocean engineering. Due to the harsh marine environment, steel corrosion is a major problem. In order to improve the corrosion resistance of steel reinforcing bars, the fiber reinforced polymer (FRP) bar, which has the characteristics of corrosion resistance and high tensile strength, has become an ideal substitute for steel bars, due to its excellent mechanical properties. However, due to its low elastic modulus, FRP-reinforced concrete structures often have large deformations, large cracks and brittle failure characteristics [1,2,3,4]. The recent development of steel–FRP composite bars (SCFCB) makes up for the lack of ductility of FRP rebars to some extent, and reduces engineering costs [5,6]. However, SCFCB is a new type of material, and related research such as flexural resistance and flexural stiffness is rare. The stress transfer mechanisms between the interfaces of steel core to FRP and SFRP to concrete are very complicated. There is no related specification available for engineers, which limits the application of SCFCB in concrete structures. Similarly, FRP-reinforced concrete research is not as mature as reinforced concrete. The related studies on FRP-reinforced concrete in different countries have significant difference [7,8,9,10,11]. Whether they can be used for different reinforced concrete need to be further clarified.

At present, research has focused on durability, flexural capacity of FRP-reinforced concrete. Studies by Chen and Kim et al. [12,13,14], show that the tensile strength of FRP bars decrease when exposed to high temperature environments. CFRP bars have no significant change when soaked in alkaline solution. However, GFRP (glass fiber reinforced polymer) bars tend to be easily degraded. In pull-out tests, it was found that the bond strength between FRP bars and concrete depends on factors such as the compressive strength of concrete, diameter of FRP bars and surface finishing [15,16,17]. Failure on the surface of the bar l will occur at temperatures higher than 60 °C [18]. Compared with reinforced beams, FRP-reinforced beams have significantly enhanced strength, but unavoidable brittle failure. The axial force of FRP, shear stress and normal stress at the bonding interface significant increases under creep [19,20]. Zhu and Wang et al. [21,22] pointed out that fiber-reinforced concrete mixed with short fibers can effectively overcome problems such as large deflection, crack width and brittle failure, especially high-strength FRP-reinforced concrete. Toutanji et al. [23] show that the formula of crack width prediction in ACI 440.1-R can accurately calculate the maximum crack width of single-layer reinforced beams with FRP bars, but the calculation results are more conservative when double-layer bars are used. The formula from existing codes overestimated flexural rigidity FRP-reinforced beams, lead to two revisions in code ACI 440.1-R.

In recent years, preliminary research has been performed on the basic mechanical properties of SCFCB and SCFCB concrete structures. Wu [24] and others carried out uniaxial tensile tests on SCFCB and found that the load-strain relationship curve showed a clear double-fold line before fiber breakage. Dong Z.Q. [25] show that after nine months of aging in SCFCB seawater at 40 °C, the bond strength decreased by 5%, and the bond strength decreased by 26.2% at 50 °C. After 50 years of service under normal temperature, the bond strength retention rate of SCFCB and SWSSC (seawater–sea sand concrete) is 84–96%. Ren, Sun [26,27,28] etc. conducted preliminary experimental research on SCFCB-reinforced concrete and tried to develop calculation formula for stiffness. In contrast, few studies have been made for SCFCB concrete structures in terms of bond slip, failure mechanism, and its difference to steel-reinforced concrete members and FRP-reinforced concrete members.

Therefore, in this study, the flexural performance of steel–carbon fiber composite bars (SCFCB) reinforced coral concrete beams is studied. A comparative study between FRP-reinforced and steel-reinforced coral concrete is also made. Their differences in terms of crack development, deflection and failure modes are discussed. The design formula from the existing codes are also examined. The applicability of the formulas of FRP-reinforced concrete to SCFCB-reinforced coral concrete beams are studied.

## 2. Test Program

Eight coral concrete beams with different types of longitudinal reinforcements were tested using four-point bending test rigs to failure. All test beams were designed according to the existing fiber reinforced concrete specifications with controlled failure due to concrete crushing in the compression zone rather than longitudinal bar rupture. The coral concrete belongs to lightweight aggregate concrete.

### 2.1. Test Materials

**Figure 1:**
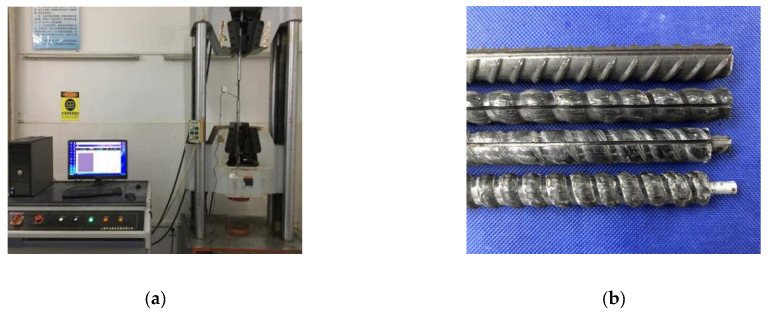
Experimental material and tensile test of reinforcement. (**a**) Tensile test of reinforcement (**b**) CFRP bars and SCFCB.

The CFRP bars used in this test were produced by Zhejiang Haining Anjie Composite Materials Co., Ltd. (Zhejiang, China) and SCFCB (one steel core was threaded steel bar and the other steel core was round high-strength steel bar) was produced by Nantong Intelligent Fiber Composite Reinforcement Co., Ltd. (Nantong, China). As the ordinary grade III rebar on the market. In order to understand the mechanical properties of bars, CFRP bar, steel bar and SCFCB conducted a uniaxial tensile test before the test (Refer to the test standard for reinforcement), as shown in Figure 1a. Among them, the bonding strength of SCFCB steel core and carbon fiber was measured by pulling out tests with the anchor length of 5 d_b_ (d_b_ was the diameter of the reinforcement). The apparent condition and basic mechanical properties of the reinforcement are shown in Figure 1b and Table 1.

The design strength of all coral concrete was C35, of which the coarse aggregate was the natural continuous graded coral debris (Figure 1a) washed up from the Beibu Gulf Channel, and the fine aggregate was coral sand (calcite sand), mixed with ordinary Portland cement (P.042.5), artificial seawater and water reducing agent. Made. At the same time as the test beam was poured, six cubic standard test pieces (150 mm × 150 mm × 150 mm) and six prism test pieces (150 mm × 150 mm × 300 mm) were produced. The test results of the basic mechanical properties of the full coral concrete after 28 days are shown in Table 2, following the test standard of ordinary concrete.

### 2.2. Test Specimens

**Figure 2:**
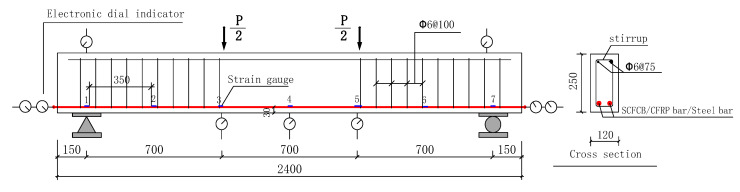
Test rigs and dimension, reinforcement detail of test beams.

The test beam was made of four different types of bars, and two test beams were made of each bar. The dimension size of the beams was 120 mm × 250 mm × 2400 mm, the thickness of the concrete cover was 25 mm, the diameter of the longitudinal rebars were 14 mm, the upper longitudinal reinforcement and the stirrups were CFRP rebars with a diameter of 6 mm. The strain gauge was used for strain measurement. The longitudinal bars at both ends of the test beam exceed the beam length by 10 mm to measure the slip between the longitudinal bars and the concrete, steel core and carbon fiber. The basic parameters of the test specimens are shown in Figure 2.

### 2.3. Loading and Instrumentation

A four-point bending test method was adopted. The load was continuously applied with 3 kN increment until failure of specimen, and the loading speed was 0.5 kN per second. The load was remained for 5 min at each stage of loading. An electronic distance meter was used to measure the width of concrete cracks at the position of the longitudinal rebars and record the crack development and speed, each reading was taken at every 9 kN increment; 7 electronic dial gauges were placed on both sides of the beam and the bottom of the beam to measure the deformation and slip, the detailed arrangement is shown in Figure 2.

## 3. Test Results and Analysis

Based on the test results, the result comparison of steel bars, carbon fiber bars, and SCFCB-reinforced coral concrete beams was made. It is worth noting that the performance of the three beams at each working stage shows obvious difference. For beams with lower concrete strength, CFRP bars cannot explore their full tensile capacity, the ultimate strength utilization of the reinforcement did not exceed 50%. The SCFCB test beam has good flexural resistance, but capacity of steel cores after yielding need to be further explored.

### 3.1. Comparison of Flexural Capacity and Failure Modes

**Figure 3:**
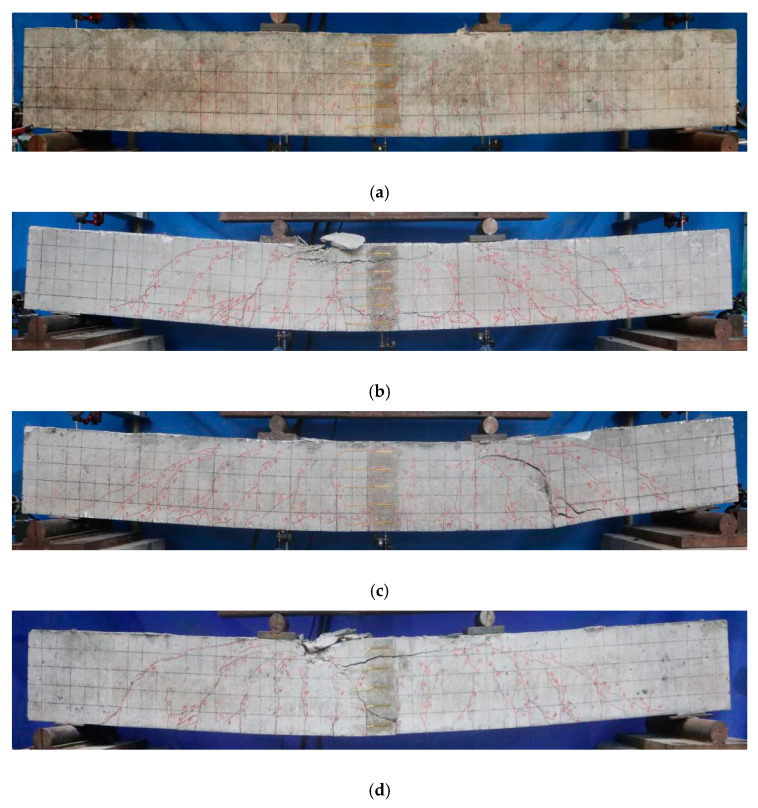
Failure pattern of specimen beams. (**a**) Rebar14-1; (**b**) CFRP14-1; (**c**) SCFCB14(6)-2; (**d**) SCFCB14(8)-2.

It can be seen from Table 3 and Figure 3 that the ultimate flexural capacity and failure modes of different types of beams show significant differences. Under the condition of the same reinforcement ratio, the SCFCB beam has a higher load capacity (The ultimate load capacity of the SCFCB test beam was between 93 kN and 120.6 kN), while the CFRP-reinforced beam and the steel-reinforced specimen beam have a capacity that was about 21% to 25% lower than that of SCFCB-reinforced beam and was relatively similar. Among them, CFRP-reinforced beams exhibit brittle failure characteristics, while SCFCB-reinforced beams and steel-reinforced beams have more ductile failure characteristics, but the failure mode of SCFCB-reinforced beams was different from that of steel-reinforced beams immediately after the beams reaching yielding point. In test SCFCB14 (6), smooth finished steel core was used, slips between steel core and fiber layer were observed after yielding. This caused shear failure of the beam. The load at yield was about 40% of the ultimate load. In comparison, for SCFCB using ribbed steel core, no obvious slip was observed, therefore, the core can continue to resist load until the concrete was damaged by compression, and the load at yield was about 52% of the ultimate load.

It was found that under the same conditions (except for the stressed longitudinal bar), large tensile strength of the bar, may not lead to greater the flexural capacity of the beam. The main reasons were: first, the lack of ductility of CFRP bars makes them fail to develop their full tensile strength, and the concrete was damaged by compression. Second, due to the use of the steel core, SCFCB has relative high modulus of elasticity and its tensile strength was 41% to 76% higher than that of steel bars, making SCFCB-reinforced beams the best in flexural capacity; third, the steel core plays an important role in SCFCB, it affects the force transmission mechanism between the steel core and fiber interface and the flexural capacity of the beams. The larger the steel content, the lower the tensile strength. The bonding strength of smooth finish steel core was lower than that of ribbed steel core, which causes the local stress loss.

### 3.2. Comparison of Load-Span Deflection Curves

**Figure 4:**
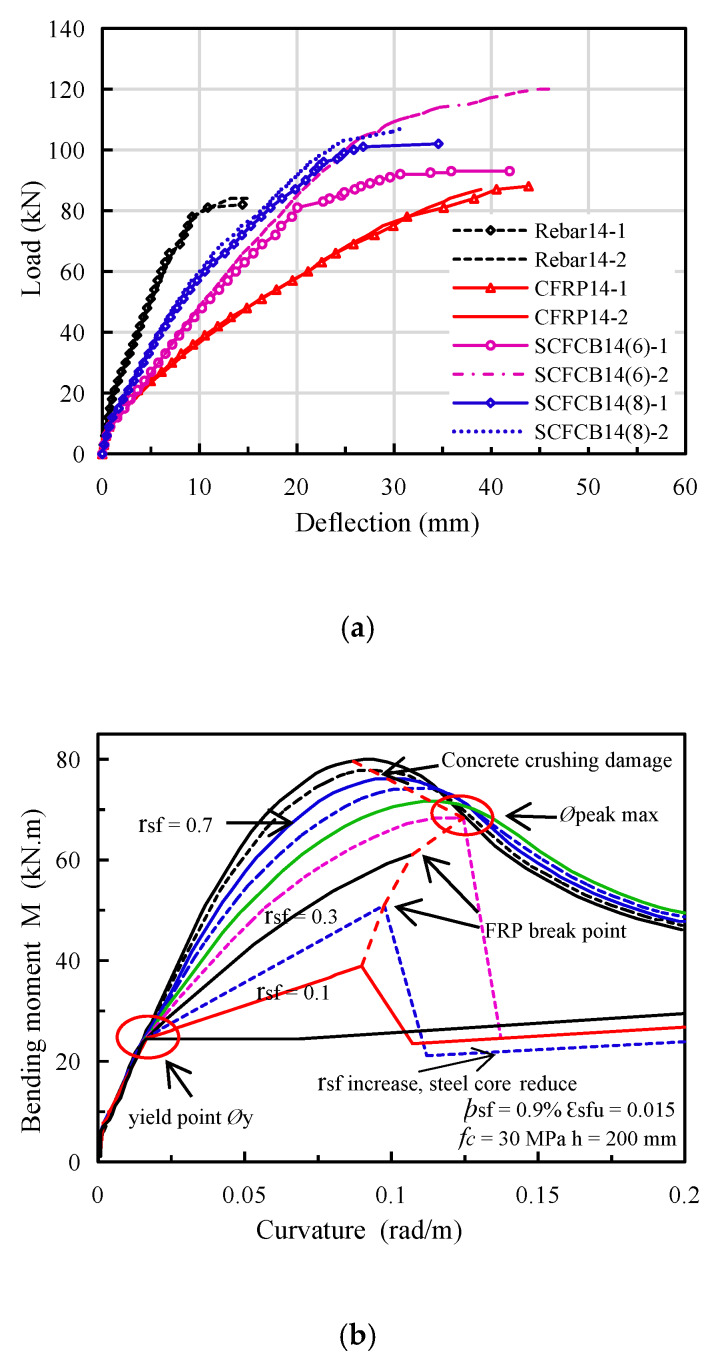

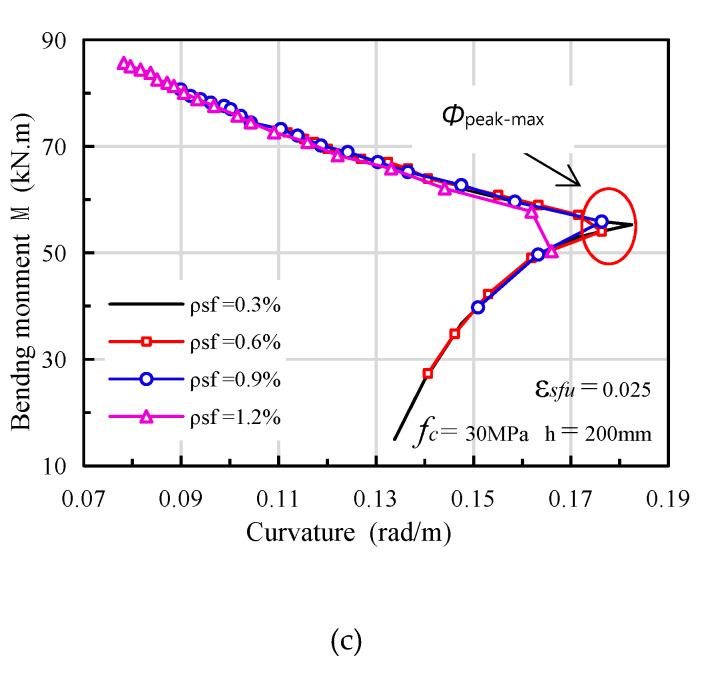
Relationship between load and mid-span deflection. (**a**) load–span mid-deflection curve. (**b**) moment–curvature of SCFCB beam [30]. (**c**) maximum possible curvature [30].

It can be found in Figure 4a that the load-deflection curves of different types of beams have obvious difference. Steel-reinforced beams exhibits 3 clear working stages: before cracking, after cracking and rebar yielding. CFRP-reinforced beams have 2 working stages, before and after concrete cracking. They were shown as double fold lines with no obvious yield stage, exhibiting brittle failure characteristics.

As a new type of composite material, the steel–fiber composite bar (SCFCB) has certain different features. It can be divided into three stages, before cracking, after cracking and steel core yielding. After the steel core yields, it will suffer a large deflection and an obvious yield stage before failure. Under the same load, the deflection of the SCFCB-reinforced beam was smaller than CFRP-reinforced beam and larger than steel-reinforced beam. For example, at 60 kN, the deflection of the SCFCB test beam was 12.97 mm, the deflection of the steel bar test beam was 6.33 mm, and the deflection of the CFRP bar test beam was 21.25 mm.

Among them, the ultimate flexural capacity of CFRP-reinforced beams was similar to that of steel-reinforced beams, but the deflection was much larger than that of the latter. For SCFCB-reinforced beams with same reinforcement ratio, the deflection of SCFCB14 (6) was greater than SCFCB14 (8). The load deflection curve showed a significant difference after the steel core yielded. There were two reasons for the above differences: first, the difference in elastic modulus (including steel content) and the specimen with a larger elastic modulus has a greater bending stiffness; second, the difference of bond due to different types of steel core surface. In the later stage of loading, the tensile stress of the smooth finish steel core was greater than the bond between the steel core and the fiber, resulting in the relative slip [29]. It was found that the tensile stress of steel core was directly related to the difference of stress transfer in fiber layer.

It should be noted that with different steel content in SCFCB, the load-deflection curve and failure mode of SCFCB-reinforced beam varied, especially after the steel core yields. Without steel core slip, the effect of steel content (fiber ratio) on the beams is shown in Figure 4b,c. When the steel content was constant, before the steel core yields, bending moment does not change significantly with the increase of the FRP ratio (r_sf_). When the fiber content was unchanged, the yielding moment increases with the increase of steel content and was subject to stress transfer and stress lag (In SCFCB test beam, when the steel core continued to be loaded at yield, the plastic deformation of the steel core increased, which damaged the bond between the steel core and the fiber, resulting in local slip of the steel core and the fiber. The fiber layer bears more stress, while the stress of the steel core was basically unchanged, but the strain of the steel core continues to increase.) impact. Therefore, the research on the flexural performance of the test beam steel core after yielding was very important for the design of SCFCB-reinforced concrete structures

### 3.3. Comparison of Crack Patterns

Generally speaking, the crack width of a flexural member was related to factors such as the thickness of the concrete cover, longitudinal reinforcement spacing, the longitudinal reinforcement diameter, the effective reinforcement ratio and the elastic modulus [30]. In all the test beams, above factors were kept the same, however, test results show that the crack width, spacing and crack pattern were quite different. Three predominant types of cracks were found: vertical cracks in pure bending zone, diagonal cracks in bending and shearing zones, longitudinal cracks in compression zones. The details are shown in Figure 3.

**Figure 5:**
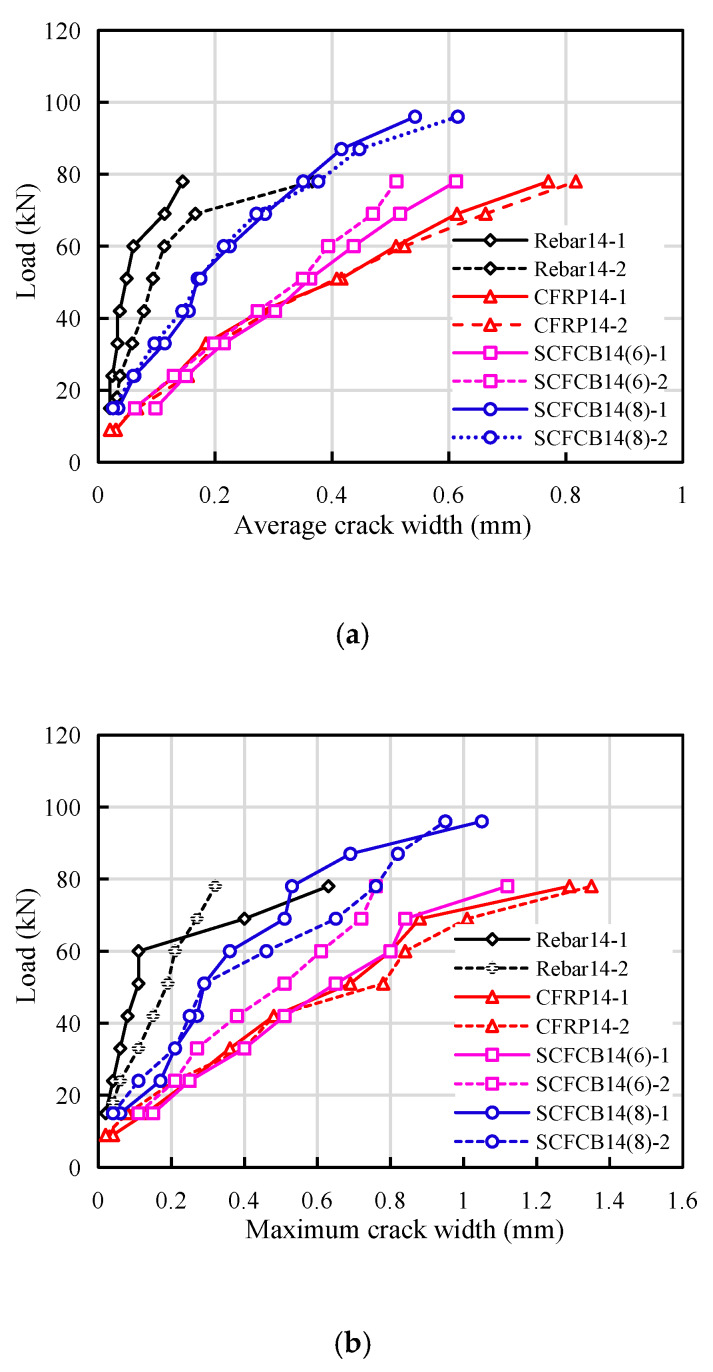
Crack width comparison from test results. (**a**) average crack width; (**b**) maximum crack width.

The cracks in steel-reinforced beams were mainly vertical and diagonal cracks, they were first observed when load increase to 9 kN and 15 kN, respectively. As shown in Figure 5, the diagonal crack with a height of 50 mm appears at 20% P_u_. the maximum crack width average crack width was still less than 0.5 mm, and the average crack spacing was 123 mm.

For CFRP-reinforced beams, the maximum crack height reached 85 mm at 9 kN. The diagonal crack appeared at a time close to that of the reinforced beam, but its development speed was much larger than that of the reinforced beam. Obvious longitudinal cracks observed at the height of the concrete at the position of longitudinal bars. The average crack height accounts for about 85% of the beam height, the maximum crack width was 1.35 mm, and the average crack spacing was about 145 mm.

For SCFCB beams, initial cracks with a height of 35–60 mm appeared at 6 kN. With the increase of the load, the crack development rate was higher than that of the steel-reinforced beam and slightly smaller than that of the CFRP-reinforced beam. When approaching failure, the longitudinal cracks were similar to CFRP beam. The largest crack width was between 1.03–1.85 mm, the average crack height accounted for about 82% of the beam height, and the average crack spacing ranged from 131 to 161 mm.

The reason for these differences was not only the difference in the elastic modulus of different types of reinforcement, but also the bond performance between the reinforcement and the concrete, as well as the steel core and the carbon fiber. The pull-out test found that the SCFCB have low bond strength values than ordinary steel bars, but similar level of strength to that of CFRP bars [29,31,32,33]. The bonding strength of the smooth finish steel core and the fiber layer was lower than that of ribbed steel core. In addition, the stress development was different between the SCFCB and CFRP rebars which also affect the crack patterns. In addition, coral concrete was lightweight and was more brittle than ordinary concrete, so obvious longitudinal cracks can be observed in the compression zone when it was close to failure.

For the bending members using new composite materials and traditional materials, the difference in their mechanical properties needs to be considered. Most FRP-reinforced concrete codes stipulate the same crack width limits as ordinary reinforced concrete. Some codes stipulate that the maximum crack width of the flexural members of fiber reinforced concrete in the indoor environment can be relaxed to 0.7 mm [7,8,9,10,11], and related research also shows that in design, FRP beams should be controlled by deflection rather than cracks [34,35,36]. However, both arguments were lack of strong experimental evidence. Compared with FRP beams, although SCFCB beams have greater bending stiffness and better ductility, their cracks, deflections and failure modes were closely related to SCFCB’s steel core percentage. These issues require further research and more data verification.

### 3.4. Comparison of the Strain Development in the Rebars

**Figure 6:**
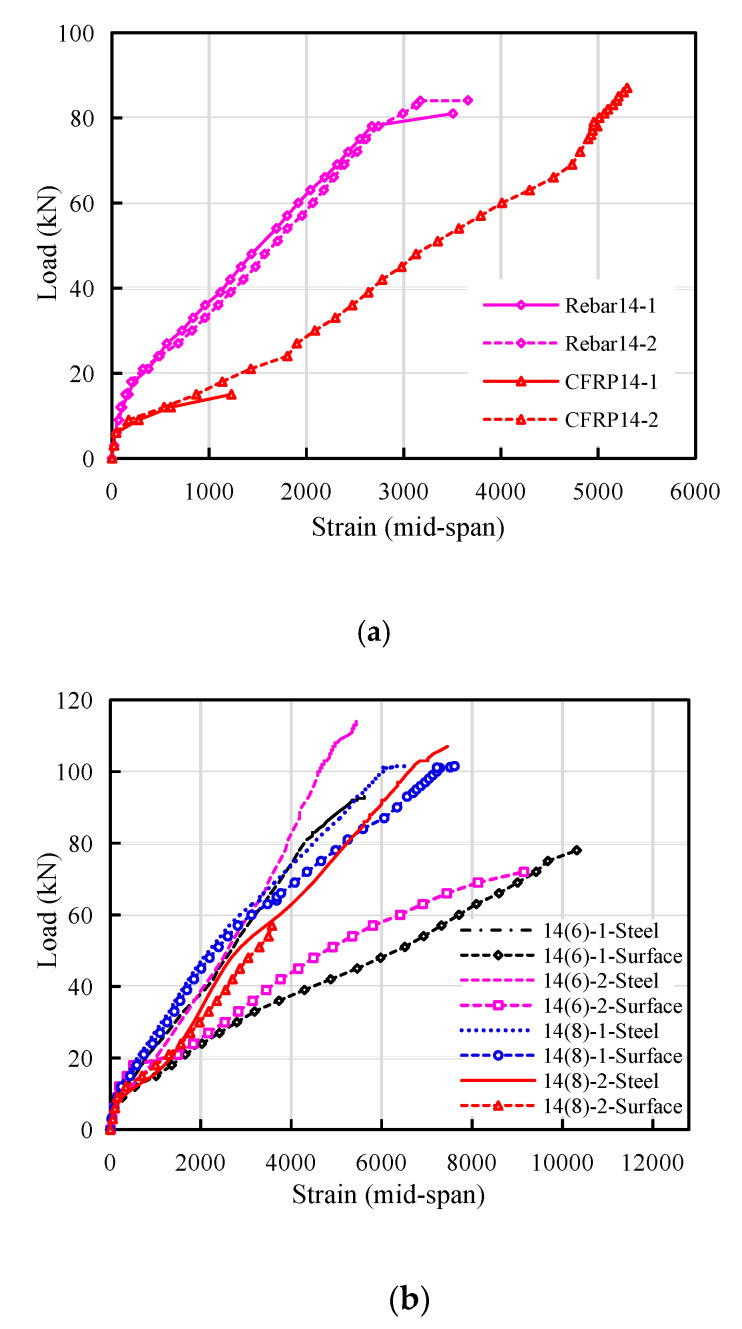
Comparison of strain in rebars. (**a**) strain comparison between CFRP and steel bar; (**b**) strain comparison between steel core and fiber.

As majority of the test beams failed in bending, the strains in the mid-span of the tensile longitudinal reinforcement were compared, as shown in Figure 6 (Strain gauge damage of CFRP14-1 specimen during loading). Under the same load, the strain of CFRP bars was much larger than that of steel bars. The former was about 1 to 2 times of the latter. The CFRP bars did not break during failure, and the maximum strain was close to 50% of its ultimate strain. Its strength utilization rate was low, while the strain of SCFCB was basically between the steel bars and CFRP bars. The maximum strain at failure exceeds the maximum strain of steel bars and CFRP bars, and its strength utilization rate was between 70% and 85%.

The comparison of the SCFCB strain in Figure 6b shows that with the increase of the load, for 14 (8), the strain development of the steel core was in phase with carbon fiber. While, for beam 14 (6), the strain of carbon fiber was twice that of the steel core. According to the analysis, higher steel content the better the performance. When the steel core and carbon fiber were not well bonded, or the steel content was low a “stress lag“ between the two material can be observed.

## 4. Suitability of Using Calculation Formulas of Different Specifications in SCFCB-Einforced Coral Concrete Structures

At present, the research on SCFCB concrete structures was still rare. Quite often, designers use design guidance for FRP-reinforced concrete structures to design SCFCB-reinforced coral concrete, it has certain limitations and deficiencies. Therefore, based on the test results from this study, this section mainly discusses the applicability of different national codes to be used in designing SCFCB-reinforced coral concrete structures.

### 4.1. Calculation and Prediction of Cracking Moment, Ultimate Bending Moment and Failure Mode

Table 4 compares the cracking moment, ultimate bending moment and failure modes in accordance with different codes with the test results. It can be seen that, most of the design standards can accurately predict the failure mode of coral concrete, but the calculation results of its flexural capacity were not ideal, it was difficult to accurately calculate the cracking moment, and overestimate the ultimate bending moment. The calculation error of the CSA specification is relatively large, and the coefficient of variation of all calculation results of the specification was more than 20%. Amr El-Nemr et al. [37] believed that the difference in the calculation of the flexural capacity of FRP-reinforced beams was due to the value of ultimate strain of concrete used in these codes. Among them, the ultimate strain of CSA [9] was 0.0035 με. Other specifications still use 0.003 με. The constitutive relationship of coral concrete, which belongs to light aggregate, was different from ordinary concrete. At present, the research of its constitutive model was not mature, and the value and influence of relevant coefficients are still difficult to determine. However, in general, the current specifications still do not pay enough attention to high tensile strength, low elastic modulus, and significantly weaker bond features of FRP bars. Therefore, the existing standard calculation formulas cannot be directly used for SCFCB coral concrete test beams. Factors such as the difference in the mechanical properties of the tendons, the bond performance and the yield strength of SCFCB should be considered.

### 4.2. Application of Crack Width and Deflection Calculation Formulas

**Figure 7:**
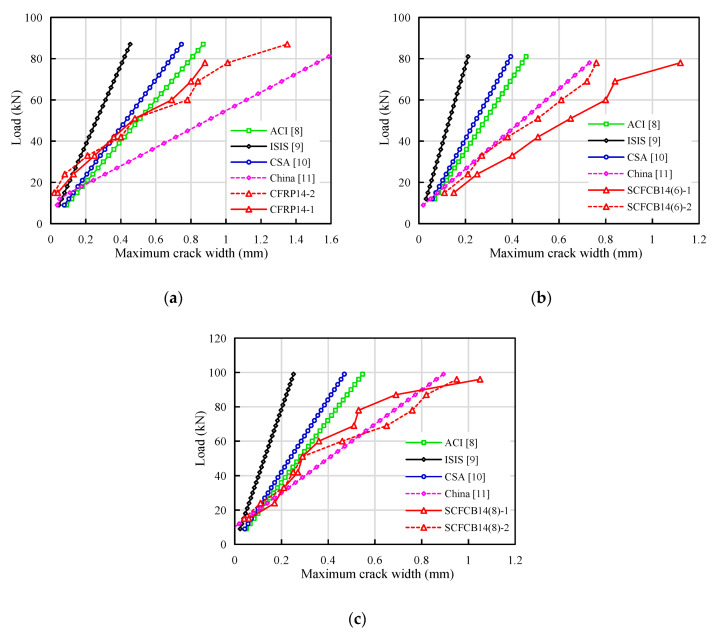
Comparison of experimental and standard calculation curves: (**a**) CFRP14 crack width calculation curve; (**b**) SCFCB14 (6) crack width calculation curve; (**c**); SCFCB14 (8) crack width calculation curve.

It can be seen from Table 5 that in the calculation of deflections and crack widths in different countries’ standard, the second modified ACI 440.1R-15 [8] deflection calculation formula was basically the same as ISIS [10]. CSA S806 [9] and China GB50608-2010 [11] were different from each other. The formula was modified based on what was proposed by Bischoff (2011) [38] with an additional factor γ to take into account the change in stiffness along the length of the member. The calculation of the crack width in most specifications consider the influence of factors such as elastic modulus, longitudinal reinforcement spacing and thickness of the concrete cover. However, the values of the bond characteristic coefficient k_b_ (vi) were still not accurate. The comparison of the calculations of the crack width of different codes are shown in Figure 7. It can be seen that it was basically difficult to accurately calculate the crack width of the coral concrete beam using any specifications. As the load increases, the error becomes more obvious. The prediction of China GB50608-2010 [11] was consistent with the test, and the error mainly comes from the variation of bond characteristic coefficient k_b_ and strain inhomogeneity coefficient ψ of the longitudinal rebars. It is worth noting that ACI 440.1R-15 [8], ISIS [10] and CSA S806 [9] calculated the maximum crack width at the bottom of the beam, while China [11] and this article calculates the maximum crack width at the height of longitudinal reinforcement. Therefore, ACI 440.1R-15 [8], ISIS [10] and CSA S806 [9] overestimates the flexural stiffness. The existing codes cannot directly used for the calculation of SCFCB-reinforced beams.

In addition, CSA S806 [10] specifies the maximum crack width as 0.50 mm when beams were used outdoors, 0.70 mm for beams used. ACI 440.1R-15 and ACI 440.1R-15 [7,8] recommends that use the value of CSA S806 [10] in most cases. ISIS [10] recommend 2000 micro strain as the strain limit of FRP bars to control the crack width. However, the test results from this study show that, when the strain of FRP bars and SCFCB (steel core and CFRP) reached 2000 micro strain, the crack width did not reach the maximum crack limit of 0.5 mm. The external load was about 30% of the ultimate load, and the SCFCB steel core was only close to yield. Therefore, the maximum crack width limit can be increased for SCFCB-reinforced concrete.

## 5. Discussion

In this paper, four-point bending tests were performed, similar test procedure and instrumentation of [39,40,41,42,43] were adopted. The suitability of using the formulas from existing codes were examined. A further discussion is made in this section. 

The reasons why the CFRP bar test beam cannot be directly calculated using the existing FRP rebar specifications were as follows: first, compared with the same strength concrete and coral concrete, the low elastic modulus and brittleness of coral concrete. Second, the bond strength between CFRP bars and coral concrete was obviously lower than that of reinforced concrete [17]. Therefore, only considering the relative bonding characteristic coefficient k_b (vi)_ was not enough to accurately reflect the overall bonding stress performance. The existing FRP bar specifications are certainly not applicable to SCFCB test beams. To establish the calculation formula of the SCFCB test beam, it was necessary to consider the stress transfer relationship between different materials and the difference in mechanical properties, as well as the change in the mechanical properties of the steel core after yielding.

In the SCFCB test beam, if the steel core yield was used as the design strength of the SCFCB, the steel content of the SCFCB should be well controlled in production or design. According to the literature [5,26,27,29] and the test results in this study, it was concluded that within a certain diameter range, the steel content of SCFCB between 30% and 56% can provide high strength utilization rate with reduced cost. When the diameter of SCFCB was large, the thickness of the fiber layer should be guaranteed to be more than three millimeters. At the same time, avoid using smooth finish steel core or steel strand to make SCFCB. Because in the high-stress state, the smooth round steel core and the fiber layers were prone to relatively slip, which makes the test beam enter the failure state early.

In SCFCB, a steel core with a certain rib height tends to have a better bonding strength with the fiber layer, while a steel core without a rib height has a poorer bonding strength with the fiber layer (the bonding strength of the two is shown in Table 1). The quality of SCFCB with smooth finish steel core was not as good as SCFCB with ribbed steel core, and the quality of SCFCB with small steel content was not as good as SCFCB with large steel content. Because the smooth finish steel core and fiber layer are prone to relative slip and steel core shrinkage under high stress, the rigidity of the test beam decreases, and the deflection and crack width increase.

## 6. Conclusions

1. The flexural capacity of the test beam was obviously different, while the bearing capacity of the SCFCB test beam was higher than the other two. At failure, SCFCB beam achieved 70% to 85% of its ultimate strength, but CFRP beams only achieved less than 50%.
2. SCFCB coral concrete beams has good ductility, but the flexural stiffness was lower than that of ordinary steel-reinforced beams and higher than that of CFRP bars reinforced beams.
3. The performance of SCFCB-reinforced beams was greatly affected by the steel core. a larger deflection can be achieved when the steel core yield.
4. SCFCB exhibits a significant “stress lag” phenomenon, which was manifested by the small and unsynchronized stress growth of the steel core, and the strain of the fiber layer was about twice the steel core strain; The steel core was related to the bonding of the fiber, and its stress transfer mechanism should be further studied.
5. When the existing FRP bar calculation specification was applied to the CFRP reinforcement coral concrete structure, there was a certain error. The bond performance between CFRP bar and coral concrete and the difference between different types of concrete should be fully considered.

## Figures and Tables

**Table 1:** Properties of bars.

Bar Type	*d_b_* (mm)	*h_h_* (mm)	*h_s_* (mm)	*f_fu_* (Mpa)	*E_f_* (GPa)	Ultimate Strain (%)		$\tau_{sf}\;(\mathrm{MPa})$
						Steel	Carbon Fiber	
Rebar 14	14	1.15	9.00	608	200.4	0.32 ± 0.03	-	-
CFRP 14	14	0.76	15.66	826	109.7	-	1.11 ± 0.03	-
SCFCB 14(6)	14	1.00	9.63	1073.8	132.6	0.31 ± 0.05	1.21 ± 0.04	3.08
SCFCB 14(8)	14	0.32	13.63	858.4	136.0	0.36 ± 0.02	1.23 ± 0.14	9.72

**Table 2:** Mechanical properties of full coral concrete.

Concrete Strength	Water–Cement Ratio	*f*_c_ (MPa)	*f_ck_* (MPa)	*f_t_* (MPa)	*E_c_* (GPa)
C35	0.33	37.7	32.5	2.10	28.8
Coefficient of variation (%)	—	0.019	0.021	0.027	0.024

**Table 3:** Cracking and failure of test beam.

Beam	ρ (%)	Steel Ratio (%)	Crack		Failure		Yield Load ^a^ (kN)	P_u_ (Kn)	Failure Mode ^b^
			P_cr_ (kN)	*w*_cr_ (mm)	*w* (mm)	*w*_mean_ (mm)			
CFRP14-1	1.18	-	9	0.02	1.29	0.77	-	88.7	CC
CFRP14-2	1.18	-	9	0.02	1.35	0.83	-	87	CC
SCFCB14(6)-1	1.18	18.39	6	0.02	1.12	0.61	45	93	SSC
SCFCB14(6)-2	1.18	18.39	6	0.02	1.85	0.9	45	120.6	SSS
SCFCB14(8)-1	1.18	32.68	6	0.02	1.05	0.54	53	102	CC
SCFCB14(8)-2	1.18	32.68	9	0.02	1.24	0.76	56	107	CC
Rebar 14-1	1.18	100	10	0.01	0.63	0.48	78	82	YCC
Rebar 14-2	1.18	100	15	0.02	0.67	0.52	78	85	YCC

Yield Load ^a^: Represents the corresponding load when the steel core (rebar) yields; Failure mode ^b^: CC: indicates that the concrete was crushed; SSC: indicates that the steel core slip and the concrete was crushed; SSS: indicates that the steel core slip and shear failure of concrete. YCC: indicates that the concrete was crushed after the steel bar yields.

**Table 4:** Experimental and predicted cracking and ultimate moments.

Beam	Experimental Results			ACI [8]			CSA [9]			ISIS [10]			GB 50608-2010 [11]		
	*M_cr_* kN·m	*M_n_* kN·m	Failure Mode ^1^	*M_cr_* Exp	*M_n_* Exp	Failure Mode ^1^	*M_cr_* Exp	*M_n_* Exp	Failure Mode ^1^	*M_cr_* Exp	*M_n_* Exp	Failure Mode ^1^	*M_cr_*^b^ Exp	*M_n_* Exp	Failure Mode ^1^
14-1	3.15	31.05	CC	1.2	0.71	CC	1.2	0.64	CC	1.2	0.75	CC	-	0.84	CC
14-2	3.15	30.45	CC	1.2	0.69	CC	1.2	0.63	CC	1.2	0.74	CC	-	0.82	CC
14(6)-1	2.10	32.55	SS^a^	0.8	0.61	CC	0.8	0.56	CC	0.8	0.65	CC	-	0.56	CC
14(6)-2	2.10	42.21	SS^a^	0.8	0.79	CC	0.8	0.73	CC	0.8	0.84	CC	-	0.73	CC
14(8)-1	2.10	35.70	CC	0.8	0.79	CC	0.8	0.74	CC	0.8	0.84	CC	-	0.75	CC
14(8)-2	3.15	37.45	CC	1.2	0.83	CC	1.2	0.78	CC	1.2	0.88	CC	-	0.78	CC
Average				1.0	0.74	-	1.0	0.68	-	1.0	0.78	-	-	0.75	-
Standard deviation				0.2	0.19	-	0.2	0.23	-	0.2	0.17	-	-	0.19	-
Coefficient of variation (%)				20%	26%	-	20%	34%	-	20%	22%	-		25%	-

^1^ CC: Concrete crushing; ^a^ SS: Steel slip; ^b^ “-”: No formula was given in the code [11].

**Table 5:** Deflection and crack width provisions.

Code	ACI Committee 440	ISIS [10]	CSA [9]	GB 50608-2010 [11]
Deflection provisions				
ACI [7]	$I_{e}={\left(\frac{M_{cr}}{M_{a}}\right)}^{3}\beta_{d}I_{g}+[1-{\left(\frac{M_{cr}}{M_{a}}\right)}^{3}]I_{cr}\leq I_{g}$ $\beta_{d}=0.2(\rho_{f}/\rho_{fb})\leq 1.0$	$I_{e}=\frac{I_{g}I_{cr}}{I_{cr}+\left(1-0.5{\left(\frac{M_{cr}}{M_{a}}\right)}^{2}\right)(I_{g}-I_{cr})}$ $\delta_{\max}=\frac{PL^{3}}{24E_{c}I_{cr}}{\left[3(\frac{a}{L})-4{\left(\frac{a}{L}\right)}^{2}-8\eta (\frac{L_{g}}{L})\right]}^{3}$	$I_{cr}=\frac{bd^{3}}{3}k^{3}+nfA_{f}d^{2}{\left(1-k\right)}^{2}$ $\delta_{\max}=\frac{PL^{3}}{24E_{c}I_{cr}}[3(\frac{a}{L})4{\left(\frac{a}{L}\right)}^{3}-8\eta {\left(\frac{L_{g}}{L}\right)}^{3}]$ $\eta =1-\frac{I_{cr}}{I_{g}}$	$\delta =\frac{6.81PL^{3}}{384B_{s}}$ $B_{s}=\frac{E_{f}A_{f}h_{of}^{2}}{1.1\psi +0.2+\frac{6\alpha_{E}\rho_{f}}{1+3.5r_{f}^{\prime }}}$ $\psi =1.3-0.74\frac{f_{tk}}{\rho_{te}\sigma_{fk}}$ $\sigma_{fk}=\frac{M_{a}}{0.9A_{f}h_{of}}$
ACI [8]	$I_{e}=\frac{I_{cr}}{1-\gamma {\left(\frac{M_{cr}}{M_{a}}\right)}^{2}[1-\frac{I_{cr}}{I_{g}}]}\leq I_{g}$			
[7,8]:	$\gamma =1.72-0.72(\frac{M_{cr}}{M_{a}})$ $\delta_{\max}=\frac{PL}{24E_{c}I_{e}}[3L^{2}-4a^{2}]$			
Crack width provision				
$w=2\frac{f_{f}}{E_{f}}\frac{h_{2}}{h_{1}}k_{b}\sqrt{d_{c}^{2}+{\left(\frac{s}{2}\right)}^{2}}$		$w=2.2k_{b}\frac{f_{f}}{E_{f}}\frac{h_{2}}{h_{1}}\sqrt[{3}]{d_{c}A}$	$w=2\frac{f_{f}}{E_{f}}\frac{h_{2}}{h_{1}}k_{b}\sqrt{d_{c}^{2}+{\left(s/2\right)}^{2}}$	$w=2.1\psi \frac{\sigma_{fk}}{E_{f}}(1.9C+0.08\frac{d_{eq}}{\rho_{te}})$ $d_{eq}=\frac{\sum n_{i}d_{i}^{2}}{\sum n_{i}v_{i}d_{i}}$

NB: in [8,9,10,11], k*_b_*(*vi*) was taken as 1.4, 1.2, 1.2, 0.7, respectively.

## Nomenclature

*A_f_*	area of FRP tension reinforcement (mm^2^)	*h_1_*	Distance from neutral axis to center of tensile reinforcement (mm)
		*h_2_*	distance from neutral axis to extreme tension fiber (mm)
*a*	shear span (mm)	*I_cr_*	moment of inertia of transformed cracked section (mm^4^)
*B_S_*	bending stiffness of beam	*I_e_*	effective moment of inertia (mm^4^)
*b*	effective beam width (mm)	*I_g_*	gross moment of inertia of uncracked section (mm^4^)
*C*	spacing or cover dimension. (mm)	*k_b_*	bond-dependent coefficient
*d*	distance from the extreme compression fiber to the centroid of tension force (mm)	*L_g_*	length of the uncracked section (mm)
		*M_cr_*	cracking moment (kN·m)
*d_b_*	bar diameter (mm)	*M_n_*	nominal moment capacity (mm)
*d_c_*	Thickness of concrete cover measured from extreme tension fiber to center of bar or wire location closest thereto (mm)	*p*	applied load (kN)
		*p_u_*	ultimate load (kN)
		*s*	spacing between the longitudinal reinforcement bars (mm)
*E_c_*	modulus of elasticity of longitudinal reinforcement (MPa)	*w*	maximum crack width (mm)
*Ef*	modulus of elasticity of longitudinal reinforcement (MPa)	*w_cr_*	Load of test beam when cracking (mm)
*f_c_*	cubic compression strength of concrete (MPa)	*w_mean_*	Average crack width (mm)
*f_ck_*	cylindric compressive strength of concrete (MPa)	ψ	coefficient of strain inhomogeneity of longitudinal bar
*f_t_*	tensile splitting strength of concrete (MPa)	$\sigma_{fk}$	stress in longitudinal reinforcement bar under specified loads (MPa)
*f_fu_*	Design tensile strength of FRP (MPa)		
*h_h_*	rib height of the bar (mm)	$\tau_{sf}$	bonding strength of steel and carbon fiber
*h_s_*	rib spacing of the bar (mm)	*p_cr_*	Load of test beam when cracking (kN)
δ	mid-span deflection (mm)	*ρ*	Longitudinal reinforcement ratio

## References

[1] Kassem C., Farghaly A.S., Benmokrance B. (2011). Evaluation of flexural behavior and service ability performance of concrete beams reinforced with FRP Bars. J. Compos. Constr..

[2] Caro M., Jemaa Y., Dirar S. (2017). Bond performance of deep embedment FRP bars epoxy-bonded intoconcrete. Eng. Struct..

[3] Zaidi A., Brahim M.M., Mouattah K., Masmoudi R. (2017). FRP properties effect on numerical deformations in FRP bars reinforced concrete elements in hot zone. Energy Procedia.

[4] Al-Sunna R., Pilakoutas K., Hajirasouliha I., Guadagnini M. (2012). Deflection behavior of FRP reinforced concrete beams and slabs: An experimental investigation. Compos. Part B Eng..

[5] Wu G., Wu Z.S., Luo Y.B., Sun Z.Y., Hu X.Q. (2010). Mechanical properties of steel-FRP composite bar under uniaxial and cyclic tensile loads. J. Mater. Civ. Eng. ASCE.

[6] Sun Z.Y., Wu G., Wu Z.S., Zhang M. (2011). Seismic Behavior of Concrete Columns Reinforced by Steel-FRP (Fiber-Reinforced Polymer) Composite Bar. J. Compos. Constr. ASCE.

[7] ACI Committee 440 (2006). Guide for the Design and Construction of Concrete Reinforced with FRP Bars (ACI 440.1R-06).

[8] ACI Committee 440 (2015). Guide for the Design and Construction of Concrete Reinforced with FRP Bars (ACI 440.1R-15).

[9] Canadian Standard Association (CSA) (2012). Design and Construction of Building Components with Fibre Reinforced Polymers.

[10] ISIS Manual No. 3 (2007). Reinforced Concrete Structures with Fifibre-Reinforced Polymers.

[11] GB 50608-2010 (2011). Technical Code for Infrastructure Application of FRP Composites.

[12] Chen Y., Davalos J.F., Ray I., Kim H.Y. (2007). Accelerated aging tests for evaluations of durability performance of FRP reinforcing bars for concrete structures. Compos. Struct..

[13] Ashrafi H., Bazli M., Najafabadi E.P., Oskouei A.V. (2017). The effect of mechanical and thermal properties of FRP bars on theirtensile performance under elevated temperatures. Constr. Build. Mater..

[14] Kim H.Y., Park Y.H., You Y.J., Moon C.K. (2008). Short-term durability test for GFRP rods under various environmental conditions. Compos. Struct..

[15] Baena M., Torres L., Turon A., Barris C. (2009). Experimental study of bond behavior between concrete and FRP bars using a pull-out test. Compos. Part B.

[16] Castel A., François R., Tourneur C. (2007). Effect of surface preconditioning on bond of carbon fiber reinforced polymer rods to concrete. Cem. Concr. Compos..

[17] Wang L., Mao Y., Lv H., Chen S., Li W. (2018). Bond properties between FRP bars and coral concrete under seawater conditions at 30, 60, and 80 °C. Constr. Build. Mater..

[18] Rezazadeh M., Carvelli V. (2018). A damage model for high-cycle fatigue behavior of bond between FRP barand concrete. Int. J. Fatigue.

[19] Mohamed H.M., Masmoudi R. (2010). Flexural strength and behavior of steel and FRP-reinforced concrete-filled FRP tube beams. Eng. Struct..

[20] Hamed E., Bradford M.A. (2012). Flexural time-dependent cracking and post-cracking behavior of FRP strengthened concrete beams. Int. J. Solids Struct..

[21] Zhu H., Cheng S., Gao D., Neaz S.M., Li C. (2018). Flexural behavior of partially fiber- reinforced high-strength concretebeams reinforced with FRP bars. Constr. Build. Mater..

[22] Wang H., Belarbi A. (2011). Ductility characteristics of fiber-reinforcedconcrete beams reinforced with FRP rebars. Constr. Build. Mater..

[23] Toutanji H., Deng Y. (2003). Deflection and crack-width prediction of concrete beams reinforced with glass FRP rods. Constr. Build. Mater..

[24] Zhishen W.G.L.Y.W., Min H.X.Z. (2010). Experimental and theoretical studies on the mechanical properties of steel-FRP composite bars. Chin. J. Civ. Eng..

[25] Dong Z., Wu G., Zhao X.L., Zhu H., Lian J. (2018). Bond durability of steel-FRP composite bars embedded in seawater sea-sand concrete under constant bending and shearing stress. Constr. Build. Mater..

[26] Ren S. (2011). Study on the Flexural Behavior of Concrete Beams Reinforced by Steel-FRP Composite Bars. Master’s Thesis.

[27] Sun Z.Y., Yang Y., Qin W.H., Ren S.T., Wu G. (2012). Experimental Study on Flexural Behavior of Concrete Beams Reinforced by Steel-Fiber Reinforced Polymer Composite Bars. J. Reinf. Plast. Compos..

[28] Seo D.W., Park K.T., You Y.J., Lee S.Y. (2016). Experimental Investigation for Tensile Performance of GFRP-Steel Hybridized Rebar. Adv. Mater. Sci. Eng..

[29] Wang L., Shen N., Zhang M., Fu F., Qian K. (2020). Bond performance of Steel-CFRP bar reinforced coral concrete beams. Constr. Build. Mater..

[30] Sun Z., Xiao W., Wu G., Yang Y., Wu Z. Preliminary Study on Moment-curvature Behavior of Concrete Beams Reinforced by Steel-Fiber Reinforced Polymer Composite Bars. Proceedings of the 9th National Construction Engineering FRP Application Academic Exchange.

[31] Zhang L. (2011). Study on Bond Behavior between Steel-FRP Composite Bar (SCFCB) and Concrete under Cyclic Loading. Ph.D. Thesis.

[32] Sun Z.Y., Wu G., Wu Z.S., Zhang J. (2014). Nonlinear Behavior and Simulation of Concrete Columns Reinforced by Steel-FRP Composite Bar. J. Bridge Eng..

[33] Xiao T.L., Qiu H.X., Li J.L. (2018). Seismic Behaviors of Concrete Beams Reinforced with Steel-FRP Composite Bars under Quasi-Static Loading. Appl. Sci..

[34] Harsha S. (2006). Tension Stiffness Effect in GFRP Reinforced Concrete Elements. Ph.D. Thesis.

[35] Adam M.A., Said M., Mahmoud A.A., Shanour A.S. (2015). Analytical and experimental flexural behavior of concrete beams reinforced with glass fiber reinforced polymers bars. Constr. Build. Mater..

[36] Aielllo M.A., Ombres L. (2000). Load-deflection analysis of FRP reinforced concrete flexural members. J. Compos. Constr..

[37] El-Nemr A., Ahmed E.A., El-Safty A., Benmokrane B. (2018). Evaluation of the flexural strength and serviceability of concrete beams reinforced with different types of GFRP bars. Eng. Struct..

[38] Bischoff P.H., Gross S.P. (2011). Equivalent Moment of Inertia Based on Integration of Curvature. J. Compos. Constr..

[39] Guo L., Liu Y., Fu F., Huang H. (2019). Behavior of axially loaded circular stainless steel tube confined concrete stub columns. Thin-Walled Struct..

[40] Gao S., Guo L., Fu F., Zhang S. (2017). Capacity of semi-rigid composite joints in accommodating column loss. J. Constr. Steel Res..

[41] Deng X.F., Liang S.L., Fu F., Qian K. (2020). Effects of High-Strength Concrete on Progressive Collapse Resistance of Reinforced Concrete Frame. J. Struct. Eng..

[42] Qian K., Liang S.L., Xiong X.Y., Fu F., Fang Q. (2020). Quasi-static and dynamic behavior of precast concrete frames with high performance dry connections subjected to loss of a penultimate column scenario. Eng. Struct..

[43] Weng Y.H., Qian K., Fu F., Fang Q. (2020). Numerical investigation on load redistribution capacity of flat slab substructures to resist progressive collapse. J. Build. Eng..

